# Microbiome-Targeted Modulation in Renal Transplantation

**DOI:** 10.3390/jcm15145648

**Published:** 2026-07-18

**Authors:** Hans Michael Hau, Nora Jahn, Robert Karitnig, Sandro Michael Hasenhütl, Robert Sucher, Philipp Stiegler, Sven Laudi

**Affiliations:** 1 Department of General, Visceral and Transplant Surgery, Medical University of Graz, 8010 Graz, Austria; robert.karitnig@medunigraz.at (R.K.); robert.sucher@medunigraz.at (R.S.);; 2 Department of Anesthesiology and Intensive Care Medicine, Medical University of Graz, 8010 Graz, Austria; nora.jahn@medunigraz.at; 3 Department of Anesthesiology and Intensive Care Medicine, University Hospital Leipzig, 04103 Leipzig, Germany; sven.laudi@medizin.uni-leipzig.de

**Keywords:** gut microbiome, renal transplantation, chronic kidney disease, gut–kidney axis, gut–liver–kidney axis, short-chain fatty acids, TMAO, bile acids, uremic toxins, fecal microbiota transplantation, probiotics, dysbiosis, immunosuppression, tacrolimus, mycophenolate mofetil, beta-glucuronidase, allograft rejection, post-transplant diabetes

## Abstract

The gut microbiome has emerged as a critical determinant of health and disease across virtually all organ systems. In the context of chronic kidney disease (CKD) and renal transplantation, mounting evidence reveals a complex bidirectional relationship between the intestinal microbiota and kidney function—commonly referred to as the gut–kidney axis. Patients with CKD harbor a profoundly altered gut microbial ecosystem characterized by reduced diversity, depletion of beneficial commensal organisms, and expansion of pathobiont taxa capable of generating uremic toxins and pro-inflammatory mediators. These perturbations are further compounded by the uremic milieu itself, dietary restrictions, frequent antibiotic exposure, and the use of immunosuppressive agents following transplantation. The gut–liver–kidney axis adds an additional layer of complexity, linking hepatic metabolism, bile acid signaling, endotoxemia, and systemic immune activation to the progression of renal disease. Gut-derived metabolites—including short-chain fatty acids (SCFAs), bile acids, trimethylamine N-oxide (TMAO), and tryptophan-derived uremic solutes such as indoxyl sulfate and p-cresyl sulfate—serve as molecular mediators of inter-organ crosstalk and have been identified as both biomarkers and therapeutic targets. A growing body of literature supports the diagnostic and prognostic utility of microbiome composition and its metabolic signatures in patients with CKD and those undergoing renal replacement therapy. Therapeutic strategies aimed at restoring microbial homeostasis—encompassing dietary interventions, prebiotics, probiotics, synbiotics, fecal microbiota transplantation (FMT), bile acid–based therapies, and novel pharmacological approaches—hold considerable promise for improving outcomes in CKD and transplant recipients. Importantly, the bidirectional relationship between immunosuppressive drugs and the gut microbiota has emerged as a clinically significant determinant of both microbial ecology and drug pharmacokinetics: each major immunosuppressive agent class—corticosteroids, calcineurin inhibitors, mycophenolate mofetil, and mTOR inhibitors—induces characteristic dysbiotic patterns, while in turn, the microbiota modulates drug bioavailability through enzymatic biotransformation (notably bacterial beta-glucuronidase activity affecting mycophenolic acid enterohepatic recirculation) and modulation of host drug-metabolizing enzymes. This narrative review provides a comprehensive overview of the current understanding of microbiome dysbiosis in the setting of renal disease and transplantation, examines the mechanistic underpinnings of the gut–liver–kidney axis, details the multifaceted impact of dysbiosis on transplant outcomes—including allograft function and rejection, infection, post-transplant diabetes, and cardiovascular complications—and critically appraises the translational potential of microbiome-targeted interventions. We conclude by highlighting ongoing challenges and future directions toward personalized, microbiome-informed clinical care.

## 1. Introduction

Chronic kidney disease (CKD) represents a major global public health burden, affecting approximately 10–13% of the adult population worldwide and accounting for substantial morbidity, mortality, and healthcare expenditure [1]. Despite advances in pharmacotherapy, dialysis technology, and transplant immunology, long-term outcomes for patients with advanced CKD and end-stage renal disease (ESRD) remain suboptimal. Renal transplantation, while offering the best prospect for improved survival and quality of life, is itself accompanied by a constellation of complications including graft rejection, opportunistic infection, metabolic derangements, and cardiovascular disease [2,3,4].

Over the past two decades, the human gut microbiome—comprising trillions of bacteria, archaea, fungi, and viruses inhabiting the gastrointestinal tract—has been recognized as a fundamental regulator of host metabolism, immune function, and gut barrier [5,6,7,8]. Perturbations in the composition and metabolic output of the gut microbiota, collectively termed dysbiosis, have been implicated in the pathogenesis of a remarkably diverse array of chronic diseases, including inflammatory bowel disease, obesity, type 2 diabetes, cardiovascular disease, and, increasingly, chronic kidney disease [9,10].

The concept of the gut–kidney axis encapsulates the bidirectional communication between the intestinal ecosystem and the kidneys, mediated by microbially derived metabolites, immune signaling pathways, and neuroendocrine mechanisms [9]. In CKD, the accumulation of uremic solutes—many of which are products of microbial metabolism—disrupts intestinal epithelial tight junctions, promotes bacterial translocation, and triggers systemic inflammation, thereby establishing a vicious cycle that accelerates renal functional decline [11,12,13]. The additional involvement of the liver in this axis, through bile acid metabolism, detoxification, and immune modulation, has given rise to the broader concept of the gut–liver–kidney axis, a tripartite framework that more fully captures the complexity of organ crosstalk in renal disease [14,15].

In the specific context of renal transplantation, the gut microbiome is subject to further profound disruption. Perioperative antibiotics, high-dose immunosuppressive regimens, and the residual effects of pre-transplant uremia conspire to reshape the intestinal microbial landscape in ways that may influence graft survival, infection susceptibility, and long-term metabolic health [16,17,18,19,20]. Emerging evidence suggests that the gut microbiome may serve not only as a modifiable risk factor but also as a source of non-invasive biomarkers for early detection of graft dysfunction and rejection [21,22,23].

The therapeutic modulation of the gut microbiome—through dietary modification, administration of probiotics, prebiotics, or synbiotics, fecal microbiota transplantation (FMT), and novel pharmacological agents—has thus emerged as a frontier of clinical investigation with the potential to transform the management of CKD and transplant recipients [24,25,26,27,28]. This narrative review aims to provide a comprehensive and critical synthesis of the current literature on the role of the gut microbiome in renal disease and transplantation, the mechanistic pathways underlying inter-organ axis dysregulation, and the translational promise and challenges of microbiome-targeted therapies.

It is important to frame the intent of this review at the outset. The evidence linking the gut microbiome to renal disease and transplant outcomes, while rapidly expanding and mechanistically compelling, remains largely preclinical and associative; robust, adequately powered clinical trials with hard endpoints are still lacking. Our aim is therefore not to issue clinical recommendations or to advocate microbiome-targeted interventions in current practice, but to shed light on an emerging and promising field—to consolidate what is presently known about the mechanisms of gut–organ crosstalk, to distinguish established findings from provisional ones, and to provide a conceptual map that can orient and prioritize future research. Any translation of these concepts into patient care will require the confirmatory, controlled evidence that the field has yet to generate.

## 2. Literature Review Approach

This article is a narrative review and does not follow a formal systematic-review protocol; it does not aim to catalog every published study, but rather to synthesize and interpret the literature that best illustrates the role of the gut microbiome in chronic kidney disease and renal transplantation. Accordingly, studies were identified and selected on the basis of the authors’ subject-matter expertise, informed by iterative searches of PubMed and by the reference lists of pertinent articles.

Searches combined terms such as “gut microbiome,” “intestinal microbiota,” “renal transplantation,” “chronic kidney disease,” “gut–kidney axis,” “gut–liver–kidney axis,” “short-chain fatty acids,” “uremic toxins,” “fecal microbiota transplantation,” “probiotics,” “prebiotics,” “synbiotics,” “dysbiosis,” “immunosuppression,” “allograft rejection,” “post-transplant outcomes,” and “microbiome therapy”. We gave particular weight to recent work and to controlled and clinical studies, while retaining seminal earlier reports and authoritative guidelines where they provided important context. Backward reference searching of key articles was used to identify additional relevant literature.

Because this is an interpretive rather than a systematic review, no formal screening algorithm, quantitative synthesis, or structured risk-of-bias appraisal was applied, and the selection of studies inevitably reflects the authors’ judgment of relevance and significance. To help the reader weigh the evidence, we indicate throughout the text the nature of the underlying data—preclinical and mechanistic, observational, or interventional—so that mechanistically plausible findings are not mistaken for clinically established ones.

**Use of AI tools:** The purpose of this review is to map an emerging and rapidly expanding field—synthesizing mechanistic evidence on gut–organ crosstalk in renal disease and transplantation and identifying directions for future research—rather than to provide clinical guidance. A body of literature this large, fast-moving, and dispersed across nephrology, hepatology, microbiology, and immunology is well suited to AI-assisted synthesis, and we therefore used such tools deliberately and transparently in service of that goal. During the preparation of this manuscript, the authors used large language models (Google Gemini 3.1 Pro, Gemini 3.5 Flash, and Anthropic Claude Opus 4.8) to assist with literature search, drafting and language editing of the text, and internal review of the manuscript. Claude Science and Consensus.app were used to support literature retrieval and scientific validation, including reference verification against the primary literature. These tools were employed as an aid to, and never a substitute for, the authors’ scholarly judgment: all AI-generated or AI-assisted content—including the text, the scientific arguments, and every reference—was subsequently reviewed, verified, and edited by the authors. Because the field remains largely preclinical and this review is explicitly interpretive rather than prescriptive, this human verification was integral to ensuring that AI assistance accelerated the synthesis of evidence without introducing unverified claims or overstating the clinical readiness of microbiome-targeted approaches. Additionally, generative AI tools were utilized to assist in designing and drawing the figures. The authors take full responsibility for the accuracy, integrity, and originality of the final manuscript.

## 3. The Gut Microbiome in Transplantation

### 3.1. Importance of the Gut Microbiota, Development, and Its Metabolites

The human gastrointestinal tract harbors an extraordinarily diverse and metabolically active microbial community that has co-evolved with its host over millennia. The gut microbiota, estimated to comprise approximately 3.8 × 10^13^ microbial cells and encoding a collective genome (the microbiome) that vastly exceeds the human genome in gene content, performs essential functions that the host cannot accomplish independently [29]. These functions include the fermentation of otherwise indigestible dietary substrates, the synthesis of essential vitamins (e.g., vitamin K, B-group vitamins), the biotransformation of bile acids, the maintenance of intestinal epithelial barrier integrity, and the education and calibration of both innate and adaptive immune responses [8,30,31,32,33,34].

The assembly of the gut microbiota begins at birth and is shaped by mode of delivery (vaginal versus cesarean), breastfeeding practices, early antibiotic exposure, and environmental factors during infancy. By approximately three years of age, the microbiota achieves a composition broadly resembling that of the adult, dominated by members of the phyla Firmicutes and Bacteroidetes, with smaller contributions from Actinobacteria, Proteobacteria, and Verrucomicrobia [35,36,37]. Throughout life, the microbiota remains responsive to dietary patterns, medication use, physical activity, and disease states, exhibiting a degree of plasticity that underlies both its vulnerability to disruption and its amenability to therapeutic intervention [38,39,40].

The metabolic output of the gut microbiota is a principal mediator of its effects on host physiology. Short-chain fatty acids (SCFAs)—primarily acetate, propionate, and butyrate—are produced through the anaerobic fermentation of dietary fiber and serve as energy substrates for colonocytes, regulators of intestinal barrier function, and modulators of systemic immune and metabolic processes [41,42,43]. Bile acids, synthesized in the liver and subjected to extensive microbial biotransformation in the intestine, function as signaling molecules through nuclear receptors (e.g., the farnesoid X receptor, FXR) and G-protein-coupled receptors (e.g., TGR5), influencing glucose and lipid metabolism, energy expenditure, and inflammatory tone [44,45,46]. Trimethylamine (TMA), generated by microbial enzymes from dietary choline, carnitine, and betaine, is oxidized in the liver to TMAO, a metabolite strongly associated with cardiovascular risk and, more recently, with CKD progression [5,47]. Tryptophan-derived metabolites, including indole, indoxyl sulfate, and kynurenine, participate in immune regulation, neuronal signaling, and, in the uremic context, contribute to endothelial dysfunction and vascular calcification [5,48,49,50].

### 3.2. Compositional Changes in Gut Microbiota in Chronic Kidney Disease and Kidney Transplant Recipients

Several landmark investigations have characterized the gut microbiome in kidney transplant recipients (KTRs) and compared it with that of patients with chronic kidney disease (CKD) and healthy controls. Ye et al. (2018) conducted a cross-sectional study of renal transplant recipients, CKD patients, and healthy subjects, demonstrating that KTRs harbor a microbiome that is distinct from both comparator groups, with intermediate alpha-diversity between CKD patients (lowest) and healthy controls (highest) [51]. Swarte et al. (2020) performed the largest single-center analysis to date, profiling the gut microbiome of 139 KTRs and identifying significant dysbiosis characterized by reduced *Faecalibacterium*, Ruminococcaceae, and Lachnospiraceae alongside enrichment of Enterobacteriaceae and Enterococcaceae [16]. Souai et al. (2020) further demonstrated that the post-transplant period and the presence of comorbid lifestyle diseases independently shape the KTR gut microbiome [52]. Lee et al. (2014) provided early pilot evidence that gut microbial community structure is associated with post-transplant complications, including infection and diarrhea [19]. Yu et al. (2021) documented significant alterations in both gut microbiota composition and circulating metabolites before and after renal transplantation, while Chan et al. (2021) examined paired live kidney donors and their recipients, reporting that donor–recipient microbial similarity may influence early graft outcomes [53,54].

The gut microbiota of patients with CKD undergoes characteristic and progressive compositional alterations that distinguish it from that of healthy individuals. Multiple cross-sectional and longitudinal studies have documented a reduction in overall microbial diversity—a hallmark of dysbiosis—accompanied by shifts in the relative abundance of specific taxa [55,56]. Broadly, CKD-associated dysbiosis is characterized by a depletion of saccharolytic, SCFA-producing genera (e.g., *Faecalibacterium*, *Roseburia*, *Bifidobacterium*, *Coprococcus*) and an expansion of proteolytic and urease-producing taxa (e.g., Enterobacteriaceae, Clostridiaceae, Desulfovibrionaceae [57]. This shift from fermentative to proteolytic metabolism results in increased generation of uremic toxins—most notably indoxyl sulfate and p-cresyl sulfate—at the expense of protective SCFA production.

The severity of microbiota disruption appears to correlate with the stage of CKD and is most pronounced in patients receiving hemodialysis or peritoneal dialysis, in whom additional factors such as dietary protein and phosphorus restrictions, oral iron supplementation, phosphate binders, and frequent antibiotic use further compound microbial [58]. Importantly, studies in animal models have demonstrated that uremia itself—independent of dietary or pharmacological confounders—is sufficient to drive gut dysbiosis, suggesting a direct pathogenic role of the uremic milieu in reshaping the intestinal ecosystem [59].

### 3.3. Gut Dysbiosis in Kidney Transplant Recipients: A Catalyst for Systemic Pathogenesis

The dysbiotic gut microbiota in CKD does not merely represent an epiphenomenon of renal dysfunction; rather, it actively participates in disease progression through multiple interconnected mechanisms. In the specific setting of kidney transplantation, the transplant procedure itself introduces an additional insult: ischemia–reperfusion injury (IRI) to the allograft generates a burst of reactive oxygen species and damage-associated molecular patterns (DAMPs) that activate innate immune responses, amplify intestinal barrier dysfunction, and exacerbate pre-existing dysbiosis [51]. Perioperative surgical stress, anesthesia, and prophylactic antibiotic administration further disrupt the intestinal microbial ecosystem during this critical early post-transplant window. Uremia-induced disruption of intestinal epithelial tight junctions increases paracellular permeability, facilitating the translocation of bacteria, endotoxins (lipopolysaccharide, LPS), and microbially derived metabolites into the systemic circulation [60]. This process, termed metabolic endotoxemia, triggers activation of innate immune pathways—including Toll-like receptor (TLR) signaling and the NLRP3 inflammasome—resulting in the sustained release of pro-inflammatory cytokines (TNF-α, IL-1β, IL-6) that perpetuate chronic systemic inflammation [61].

Several key drivers of gut dysbiosis in CKD warrant emphasis. Uremia exerts direct toxic effects on the intestinal epithelium and alters luminal pH and substrate availability, favoring the expansion of pathobiont taxa. Medications commonly prescribed in CKD—including proton-pump inhibitors, oral iron preparations, phosphate binders, and antibiotics—independently alter microbial composition. Dietary restrictions, particularly limitations on fiber, fruit, and vegetable intake imposed to control potassium and phosphorus levels, deprive commensal bacteria of the fermentable substrates required for SCFA production, further exacerbating dysbiosis [62]. Environmental factors, including reduced physical activity and psychosocial stress, may also contribute to microbial imbalance, although these influences remain less well characterized in the CKD population.

## 4. The Gut–Kidney, Gut–Liver, and Liver–Kidney Axes

**Figure 1:**
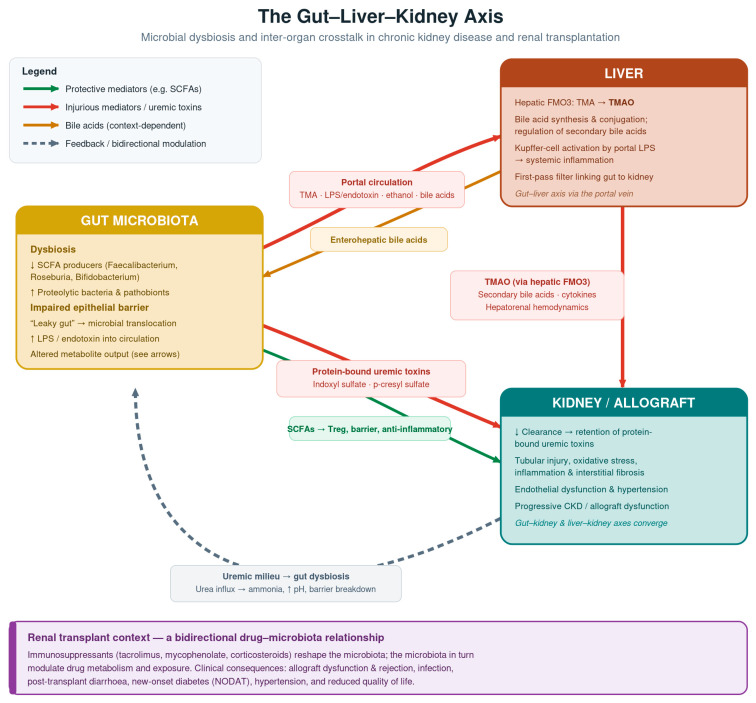
The gut–liver–kidney axis in chronic kidney disease and renal transplantation. Gut dysbiosis and impaired intestinal-barrier integrity increase lipopolysaccharide (LPS) translocation and alter microbial metabolite output. Trimethylamine (TMA), endotoxin, and bile acids reach the liver through the portal circulation, where TMA is converted to trimethylamine-N-oxide (TMAO) by hepatic flavin-containing mono-oxygenase 3 (FMO3) and Kupffer cells are activated. Protein-bound uremic toxins (indoxyl sulfate, p-cresyl sulfate), TMAO, secondary bile acids, and inflammatory mediators promote tubular injury, inflammation, interstitial fibrosis, and endothelial dysfunction in the kidney, whereas short-chain fatty acids (SCFAs) exert counter-regulatory, protective effects. Declining renal clearance generates a uremic milieu that further aggravates dysbiosis, closing a self-reinforcing loop. In transplant recipients, immunosuppressive drugs and the microbiota interact bidirectionally, with consequences for allograft function and rejection, infection, post-transplant diarrhea, new-onset diabetes after transplantation (NODAT), hypertension, and quality of life.

The major mechanism underlaying the gut-kidney, gut-liver and liver-kidney axes involved in CKD and renal transplantation are summarized in Figure 1. 

### 4.1. Gut–Kidney Axis: Microbial Toxins and Renal Responses

The gut–kidney axis describes the bidirectional interaction between the intestinal microbiota and the kidneys, whereby microbial metabolites influence renal physiology and, conversely, kidney function modulates the intestinal environment [9]. In health, the kidneys efficiently clear microbially derived solutes such as indoxyl sulfate, p-cresyl sulfate, and TMAO. As renal function declines, however, these uremic toxins accumulate in the circulation, exerting direct nephrotoxic effects that include promotion of tubulointerstitial fibrosis, oxidative stress, and inflammation within the renal parenchyma [63]. Indoxyl sulfate, derived from microbial tryptophan metabolism, activates the aryl hydrocarbon receptor (AhR) and nuclear factor kappa B (NF-κB) pathways in renal tubular epithelial cells, promoting epithelial-to-mesenchymal transition, extracellular matrix deposition, and progressive fibrosis [64]. p-Cresyl sulfate, arising from microbial tyrosine and phenylalanine metabolism, similarly induces oxidative stress and apoptosis in proximal tubular cells. TMAO has been shown to activate renal fibrosis pathways and to promote vascular calcification, a major contributor to cardiovascular mortality in CKD [65]. Simultaneously, the failing kidney contributes to gut dysbiosis by allowing urea and other uremic solutes to diffuse into the intestinal lumen, where they are metabolized by urease-producing bacteria, thereby generating ammonia and further disrupting epithelial barrier function.

### 4.2. Gut–Liver Axis: Metabolic and Immune Interactions

The gut–liver axis represents the intimate anatomical and functional relationship between the intestine and the liver, connected via the portal venous system, which delivers gut-derived nutrients, microbial products, and immune signals directly to the hepatic sinusoids [66]. Under physiological conditions, the liver serves as a firewall, clearing endotoxins and bacterial metabolites via Kupffer cell–mediated phagocytosis and hepatocyte-mediated detoxification. Intestinal barrier dysfunction, as occurs in CKD-associated dysbiosis, results in increased portal delivery of LPS and other pathogen-associated molecular patterns (PAMPs), overwhelming hepatic clearance capacity and triggering hepatic and systemic inflammation [67].

Endotoxemia activates hepatic innate immune cells, promoting the release of pro-inflammatory cytokines and acute-phase reactants, and driving a state of chronic immune activation that extends beyond the liver to affect distant organs, including the kidneys and vasculature. Furthermore, dysbiosis-induced alterations in bile acid metabolism—characterized by an increase in primary bile acids and a decrease in secondary bile acid species—disrupt FXR and TGR5 signaling, with downstream consequences for glucose homeostasis, lipid metabolism, and intestinal immune regulation [46]. The liver also plays a central role in the metabolism of gut-derived uremic toxins: indoxyl sulfate and p-cresyl sulfate undergo hepatic sulfation, while TMA is oxidized to TMAO by hepatic flavin-containing monooxygenases (FMO3). Hepatic dysfunction, whether primary or secondary to systemic inflammation, may therefore impair the processing of these toxins, amplifying their nephrotoxic and cardiovascular effects.

### 4.3. Liver–Kidney Axis: Hemodynamic, Metabolic, and Inflammatory Crosstalk

The liver and kidneys are linked by shared hemodynamic, metabolic, and inflammatory pathways that are increasingly recognized as contributors to the pathogenesis of both hepatorenal and cardiorenal syndromes [68]. Hepatic cirrhosis, for example, induces splanchnic vasodilation and systemic underfilling, leading to renal vasoconstriction and the clinical syndrome of hepatorenal syndrome (HRS). Conversely, CKD-associated uremia promotes hepatic steatosis, oxidative stress, and fibrogenesis through the accumulation of gut-derived uremic toxins and chronic inflammatory mediators. Shared metabolic pathways, including the metabolism of bile acids, branched-chain amino acids (BCAAs), and lipids, further integrate hepatic and renal function in health and disease.

### 4.4. Integrated Pathological Framework: Three Core Mechanisms Driving Axis Dysregulation

The dysregulation of the gut–liver–kidney axis in CKD and renal transplantation can be distilled into three core, interdependent mechanisms. First, intestinal barrier dysfunction permits the translocation of microbial products and toxins, initiating systemic immune activation. Second, the altered metabolic output of the dysbiotic microbiota—characterized by increased uremic toxin generation and decreased SCFA production—directly injures both the kidneys and the liver while perpetuating gut inflammation. Third, chronic systemic inflammation, sustained by endotoxemia and uremic toxin accumulation, drives organ fibrosis, vascular calcification, and immune dysregulation, creating a self-reinforcing pathological loop that accelerates disease progression [10].

## 5. Gut-Derived Metabolites, the Gut–Liver–Kidney Axis, and Microbiome-Derived Biomarkers

### 5.1. Short-Chain Fatty Acids (SCFAs)

Short-chain fatty acids—principally acetate, propionate, and butyrate—are the primary end products of anaerobic microbial fermentation of dietary fiber in the colon and represent some of the most extensively studied mediators of microbiome–host interactions. Butyrate serves as the preferred energy source for colonocytes and is essential for the maintenance of intestinal epithelial barrier integrity through its effects on tight junction protein expression and mucin secretion [69]. Beyond the gut, SCFAs exert systemic anti-inflammatory and immunomodulatory effects via activation of G-protein-coupled receptors (GPR41, GPR43, GPR109A) and inhibition of histone deacetylases (HDACs), modulating regulatory T cell differentiation, cytokine production, and macrophage function [43].

In CKD, the depletion of SCFA-producing bacteria—a consistent finding across studies—results in diminished colonic SCFA concentrations and a loss of their protective effects on the gut barrier and systemic immune tone. Supplementation with dietary fiber or direct SCFA administration has been shown in preclinical models to attenuate renal inflammation, reduce proteinuria, and slow the progression of fibrosis. Clinical studies, while limited, suggest that increased dietary fiber intake is associated with improved estimated glomerular filtration rate (eGFR) and reduced circulating levels of uremic toxins in CKD patients [70].

### 5.2. Bile Acids

Bile acids are synthesized from cholesterol in the liver and secreted into the duodenum, where they facilitate dietary lipid absorption. In the distal ileum and colon, gut bacteria extensively modify bile acids through deconjugation, dehydroxylation, and epimerization reactions, generating a diverse pool of secondary bile acids with distinct signaling properties. The bile acid pool composition modulates FXR and TGR5 signaling in the intestine, liver, kidneys, and other tissues, influencing glucose and lipid metabolism, energy expenditure, and inflammatory responses [46].

In CKD, an altered gut microbiome leads to disrupted bile acid metabolism, with a relative increase in conjugated primary bile acids and a decrease in secondary bile acid species. These changes diminish FXR activation in the intestine, leading to increased intestinal permeability, reduced expression of antimicrobial peptides, and enhanced bacterial translocation. In the kidney, impaired bile acid signaling may promote tubulointerstitial injury and fibrosis. Bile acid–based therapeutic strategies, including FXR agonists (e.g., obeticholic acid) and TGR5 agonists, are under investigation for their potential to restore bile acid homeostasis and mitigate renal and hepatic injury in CKD [71].

### 5.3. Trimethylamine N-Oxide (TMAO)

TMAO is a small organic molecule generated through a two-step process: gut microbial enzymes (TMA lyases) cleave dietary choline, phosphatidylcholine, L-carnitine, and betaine to produce trimethylamine (TMA), which is subsequently oxidized to TMAO by hepatic FMO3.73 Elevated circulating TMAO levels have been consistently associated with increased cardiovascular risk in the general population and, more recently, with accelerated CKD progression and adverse outcomes in transplant recipients.

In CKD, impaired renal clearance of TMAO leads to its systemic accumulation, where it promotes endothelial dysfunction, platelet hyperreactivity, foam cell formation, vascular inflammation, and renal fibrosis. Large-scale cohort studies have demonstrated that plasma TMAO concentrations independently predict future risk of incident CKD, progression to ESRD, and mortality, with effect sizes comparable to or exceeding those of traditional risk factors such as diabetes and hypertension [72]. Strategies to reduce TMAO levels—including dietary modification (reduction of red meat and egg consumption), targeted inhibition of microbial TMA lyases (e.g., 3,3-dimethyl-1-butanol, DMB), and FMO3 modulation—are actively being explored as therapeutic interventions.

### 5.4. Tryptophan-Derived Metabolites

Tryptophan, an essential amino acid obtained exclusively from the diet, is metabolized by the gut microbiota through several pathways yielding biologically active metabolites with relevance to renal disease. The indole pathway, mediated by bacterial tryptophanase, produces indole, which is absorbed and converted to indoxyl sulfate via hepatic cytochrome P450 enzymes and sulfotransferases. Indoxyl sulfate is a prototypical protein-bound uremic toxin that accumulates progressively as renal function declines and is not efficiently removed by conventional hemodialysis [63].

Indoxyl sulfate activates AhR and NF-κB signaling in renal tubular cells, endothelial cells, and vascular smooth muscle cells, promoting oxidative stress, inflammation, endothelial dysfunction, and vascular calcification. p-Cresyl sulfate, derived from microbial metabolism of tyrosine and phenylalanine, similarly induces tubular cell injury, inhibits endothelial cell proliferation, and is independently associated with cardiovascular events and all-cause mortality in CKD patients [73]. Kynurenine, another tryptophan metabolite generated via the host indoleamine 2,3-dioxygenase (IDO) pathway, is upregulated in the context of inflammation and has been linked to immune dysregulation and vascular disease in uremia.

These tryptophan-derived metabolites are increasingly recognized as clinically relevant biomarkers. Elevated plasma levels of indoxyl sulfate and p-cresyl sulfate correlate with CKD stage, cardiovascular morbidity, and mortality, and their reduction through dietary intervention, adsorbent therapy (e.g., AST-120), or microbiome modulation represents a therapeutic priority [74].

### 5.5. Other Metabolites

Branched-chain amino acids (BCAAs—leucine, isoleucine, valine) are metabolized by both the host and the gut microbiota, and altered BCAA metabolism has been observed in CKD and metabolic syndrome. Microbially derived phenylacetylglutamine (PAGln) has recently emerged as an independent predictor of cardiovascular events. Hippuric acid, derived from microbial benzoate metabolism, accumulates in uremia and may contribute to tubular injury. The expanding catalog of microbiome-derived metabolites with putative roles in renal disease underscores the richness of the gut–kidney metabolic interface and the potential for discovery of novel biomarkers and therapeutic targets.

### 5.6. Diagnostic and Prognostic Utility of Gut Microbiome Composition

The distinctive microbial signatures associated with CKD, diabetic kidney disease (DKD), and transplant rejection have stimulated interest in the development of microbiome-based diagnostic and prognostic tools. Metagenomic and metabolomic profiling of stool and blood samples has revealed compositional and functional alterations that discriminate CKD patients from healthy controls with reasonable accuracy [75]. Specific microbial taxa and metabolite panels have been proposed as biomarkers for CKD progression, cardiovascular risk stratification, and early detection of allograft dysfunction.

In renal transplantation, preliminary studies suggest that shifts in gut microbiome composition—particularly a reduction in diversity and an increase in Proteobacteria—may precede clinical episodes of acute rejection, raising the prospect of non-invasive microbiome-based surveillance [23]. Similarly, urinary and plasma metabolomic signatures enriched in microbially derived solutes may complement histological and immunological assessments for monitoring graft health. While these approaches remain largely investigational, ongoing large-scale prospective studies are expected to clarify their clinical validity and utility.

## 6. The Impact of Gut Microbiota Dysbiosis on Kidney Transplantation

### 6.1. Uremic Retention Solutes

Even following successful kidney transplantation, a substantial proportion of patients maintain elevated levels of gut-derived uremic retention solutes, reflecting the persistence of transplant-associated dysbiosis. The prototypical protein-bound toxins indoxyl sulfate (IS) and p-cresyl sulfate (p-CS) originate from bacterial catabolism of aromatic amino acids in the colon: tryptophan is converted to indole by bacterial tryptophanase and subsequently to IS via hepatic cytochrome P450 enzymes and sulfotransferases, while tyrosine and phenylalanine are metabolized by proteolytic bacteria to p-cresol and thence to p-CS. Trimethylamine N-oxide (TMAO), generated through microbial TMA lyase activity on dietary choline and L-carnitine, is an additional toxin whose renal clearance remains impaired in transplant recipients with suboptimal graft function [76,77].

The dysbiotic signature of kidney transplant recipients (KTRs) is characterized by enrichment of urease- and proteolytic enzyme-expressing taxa—notably Enterobacteriaceae, Proteobacteria, and Enterococcaceae—alongside depletion of short-chain fatty acid (SCFA)-producing genera including *Faecalibacterium prausnitzii*, *Roseburia*, Lachnospiraceae, and Ruminococcaceae [51,76]. This compositional shift drives overproduction of uremic toxin precursors and sustained luminal urease activity, particularly as residual uremia (even in recipients with stable graft function) continues to fuel urea diffusion into the intestinal lumen and promote pathobiont expansion [16,78,79]. Circulating IS and p-CS levels in KTRs are independently associated with accelerated decline in estimated glomerular filtration rate (eGFR), cardiovascular events, and all-cause mortality—underscoring the continued nephrotoxic relevance of these metabolites beyond initial transplantation.

### 6.2. Allograft Function

The composition of the gut microbiota has emerged as an independent determinant of early and long-term allograft function. Longitudinal studies demonstrate that greater pre- and peri-transplant microbial diversity is associated with more favorable early post-transplant eGFR trajectories and a lower incidence of infectious complications within the first six months after transplantation [51]. Mechanistically, dysbiosis-driven translocation of lipopolysaccharide (LPS) and bacterial metabolites across a compromised intestinal epithelial barrier activates TLR4–NF-κB and MyD88-dependent innate immune pathways, generating a pro-inflammatory milieu—rich in TNF-α, IL-6, and IL-1β—that directly injures the transplanted kidney through oxidative stress, endothelial dysfunction, and promotion of tubulointerstitial fibrosis [51,80,81].

Swarte et al. (2025) documented that reductions in alpha-diversity and depletion of *Faecalibacterium* persist for up to two decades after transplantation, establishing dysbiosis as a durable rather than transient phenomenon [78]. This long-term microbial imbalance sustains low-grade endotoxemia and uremic toxin burden that cumulatively impairs graft function over years. Accumulation of IS and p-CS promotes epithelial-to-mesenchymal transition and extracellular matrix deposition in proximal tubular cells via aryl hydrocarbon receptor (AhR) and NF-κB signaling, while TMAO activates renal fibrosis pathways and vascular calcification mechanisms relevant to chronic allograft nephropathy [14,20,82].

### 6.3. Allograft Rejection

The gut microbiome exerts profound influence over alloreactive and tolerogenic immune responses, with dysbiosis predisposing transplant recipients to acute rejection through multiple immunological mechanisms. Under physiological conditions, commensal organisms such as *Faecalibacterium prausnitzii*, *Prevotella*, and members of the Lachnospiraceae family produce butyrate and other SCFAs that promote the differentiation of FoxP3+ regulatory T cells (Tregs) and the secretion of anti-inflammatory IL-10, fostering a tolerogenic immune milieu that mitigates allograft recognition [76,81]. In the dysbiotic post-transplant gut, depletion of these SCFA-producing bacteria reduces butyrate concentrations, thereby impairing HDAC inhibition and histone acetylation in T cells and dendritic cells, and shifting the balance from Treg induction toward Th1 and Th17 polarization.

Expansion of pathobionts such as *Escherichia coli* and *Clostridium* difficile generates LPS and other pathogen-associated molecular patterns (PAMPs) that activate dendritic cells, promoting Th17 differentiation and IL-17 production. IL-17 upregulates endothelial adhesion molecules (ICAM-1, VCAM-1), amplifies leukocyte recruitment to the graft, and drives tissue inflammation in a pattern that is histologically consistent with acute cellular [81]. Pre-transplant microbial community profiles have been identified as significant predictors of subsequent rejection risk, and dysbiosis-associated reduction in microbial diversity may precede clinical episodes of acute rejection, raising the prospect of microbiome-based surveillance strategies for non-invasive monitoring of graft immunological status [51,83].

### 6.4. Immunosuppression Metabolism

An under-recognized consequence of post-transplant dysbiosis is its capacity to alter the pharmacokinetics of the immunosuppressive drugs upon which graft survival depends. Dysbiosis modulates the expression and activity of intestinal drug-metabolizing enzymes—most notably CYP3A4—through at least two mechanisms: LPS-mediated TLR4 activation suppresses CYP3A4 transcription via NF-κB and JAK-STAT signaling, while dysbiosis-driven reductions in SCFA production impair the epigenetic regulation of enzyme [76]. Simultaneously, barrier dysfunction alters P-glycoprotein localisation and function, affecting drug efflux and absorption. These changes produce clinically meaningful alterations in the blood levels of calcineurin inhibitors such as tacrolimus and cyclosporine, which have narrow therapeutic windows and are extensively metabolized by CYP3A4 and transported by P-gp [83,84].

Of particular pharmacological significance is the effect of microbial beta-glucuronidase (GUS) activity on mycophenolate mofetil (MMF) metabolism. Mycophenolic acid (MPA), the active moiety of MMF, undergoes hepatic glucuronidation to form the inactive mycophenolic acid glucuronide (MPAG), which is secreted in bile and enters the colon, where bacterial GUS deconjugates MPAG back to active MPA—a process that sustains enterohepatic recirculation and accounts for approximately 10–60% of total MPA systemic exposure. Dysbiosis, particularly that induced by MMF itself (see Section 6.1), depletes GUS-producing commensals including *Bacteroides* and *Faecalibacterium prausnitzii*, thereby reducing MPAG deconjugation, impairing MPA bioavailability, and potentially contributing to therapeutic failure. Zhang et al. (2021) evaluated 97 kidney transplant recipients and found that post-transplant diarrhea was associated with lower gut microbial diversity and reduced relative abundance of 12 genera [85]. In a subset of that cohort, higher fecal β-glucuronidase activity was associated with a more prolonged course of post-transplant diarrhea, with diarrhea lasting ≥7 days in 91% of patients with higher activity versus 40% of those with lower activity. These findings support a link between commensal bacterial metabolism and gastrointestinal MMF toxicity, and suggest fecal β-glucuronidase activity as a potential biomarker rather than a confirmed clinical test [81,84,85].

### 6.5. Post-Transplant Infection

Infections remain the leading cause of morbidity and the second leading cause of death in the first year after kidney transplantation, and a growing body of evidence implicates gut dysbiosis as a principal driver of infection susceptibility. The mechanisms are multiple and reinforcing: dysbiosis-driven reduction in SCFA production impairs intestinal epithelial tight junction protein expression (ZO-1, claudin-1, occludin), increasing paracellular permeability; concurrent depletion of antimicrobial peptide-producing commensals (*Bifidobacterium*, *Lactobacillus*) removes an important line of mucosal defense; and LPS-mediated chronic endotoxemia leads to progressive immune cell exhaustion with diminished pathogen surveillance [51,77,86].

Systematic review evidence confirms that both kidney and liver transplant recipients consistently exhibit enrichment of opportunistic pathogens—including Enterobacteriaceae, Enterococcaceae, Fusobacteriaceae, and Streptococcaceae—in 10 of 13 clinical studies of post-transplant microbiota [87]. In the urinary tract, expansion of Enterobacteriaceae and Enterococcus populations in a dysbiotic gut provides a reservoir for recurrent urinary tract infections (UTIs), which are the most common post-transplant bacterial infections [76]. The oral microbiome of transplant recipients similarly undergoes dysbiotic change, with documented increases in Klebsiella pneumoniae, Pseudomonas fluorescens, Acinetobacter spp., and Candida spp., contributing to opportunistic stomatitis, pneumonia, and systemic fungal infections [23]. Metagenomically, urinary cell-free DNA sequencing has demonstrated the presence of BK polyomavirus, JC virus, adenovirus, and parvovirus in transplant recipients before clinical viremia is detectable, supporting a role for altered mucosal immune surveillance in viral infection risk [84].

### 6.6. Post-Transplant Diarrhea

Post-transplant diarrhea (PTD) is a prevalent and debilitating complication, affecting 20–50% of renal transplant recipients and frequently necessitating dose reduction or substitution of immunosuppressive regimens with potential consequences for graft survival. Gut dysbiosis occupies a central mechanistic role in PTD through several overlapping pathways. Dysbiosis-associated depletion of Lachnospiraceae and Ruminococcaceae reduces colonic butyrate production, impairing epithelial cell energy metabolism, tight junction integrity, and the chloride secretion-reabsorption balance, thereby promoting a secretory diarrhoeal [51].

The relationship between MMF, gut bacteria, and PTD is particularly instructive. Landmark preclinical studies demonstrated that germ-free and antibiotic-pretreated mice are protected from MMF-associated gastrointestinal toxicity, definitively implicating the gut microbiota in this complication [84]. Bacterial GUS activity deconjugates MPAG to liberate active MPA within the colonic lumen, and high luminal MPA concentrations inhibit the proliferation of rapidly dividing intestinal epithelial crypt cells—directly impairing mucosal renewal and barrier function. Higher fecal GUS activity, positively correlated with Coprococcus and Subdoligranulum abundance, was associated with prolonged diarrhea duration in a clinical cohort of 97 KTRs, demonstrating that GUS-active microbiota amplify MMF-related gastrointestinal toxicity [84,85]. *Clostridium* difficile infection, facilitated by antibiotic-induced dysbiosis and disruption of colonization resistance, is an additional common cause of PTD in immunosuppressed recipients. Early clinical experience with fecal microbiota transplantation (FMT) for refractory PTD in transplant recipients has been encouraging, though safety considerations in the immunocompromised host require careful evaluation [76,81].

### 6.7. New-Onset Diabetes After Transplantation (NODAT)

New-onset diabetes after transplantation (NODAT) complicates 15–20% of renal transplantations and is associated with significantly increased risks of graft loss, cardiovascular events, and mortality. The gut microbiome has emerged as an important pathophysiological link between the post-transplant metabolic environment and the development of insulin resistance and beta-cell dysfunction. Dysbiotic signatures associated with NODAT in transplant recipients include depletion of Prevotella-9 and SCFA-producing genera (*Clostridium* clusters IV and XIVa, *Roseburia*, *Eubacterium*), accompanied by enrichment of *Escherichia-Shigella* and *Subdoligranulum*—a microbial pattern that mirrors the dysbiotic signature of type 2 diabetes in non-transplant populations [14,76,82].

The mechanistic pathways are interrelated. Dysbiosis-driven LPS translocation activates TLR4-NLRP3 inflammasome signaling, generating IL-1β and IL-18, which are potent inducers of systemic insulin resistance and impair pancreatic beta-cell function—effects compounded by the direct diabetogenic properties of tacrolimus and [81,88]. Reduced SCFA production impairs GPR43- and GPR41-mediated enteroendocrine signaling, decreasing GLP-1 secretion and glucose-stimulated insulin secretion. Simultaneously, dysbiosis-associated alterations in secondary bile acid metabolism—with reduction in TGR5-activating secondary bile acids—further impair GLP-1 release and glucose homeostasis. Elevated TMAO levels, a consequence of TMA-producing pathobiont expansion, independently promote mitochondrial dysfunction and metabolic endotoxemia, compounding the metabolic burden. Notably, depletion of Prevotella-9 has been proposed as a candidate microbiome-based biomarker for NODAT risk stratification in the pre-transplant assessment, warranting prospective validation [76].

### 6.8. Inflammation

Chronic low-grade systemic inflammation is a hallmark of the post-transplant state and a driver of graft dysfunction, cardiovascular disease, and impaired immune homeostasis. Gut dysbiosis perpetuates this inflammatory state through a self-reinforcing cascade. Barrier dysfunction permits translocation of LPS and other PAMPs into the portal and systemic circulation, activating TLR4 on monocytes, macrophages, and dendritic cells via MyD88-dependent signaling to produce TNF-α, IL-1β, IL-6, IL-8, and IL-23. NLRP3 inflammasome activation adds IL-18 to this cytokine milieu, while dysbiosis-reduced IL-10 production (from impaired Treg differentiation) removes a critical counter-regulatory brake [51,81].

Circulating IS and p-CS further amplify inflammation through AhR ligation and NF-κB activation in renal tubular epithelial cells, endothelial cells, and vascular smooth muscle cells, generating reactive oxygen species (ROS), promoting endothelial dysfunction, and stimulating expression of adhesion molecules that facilitate leukocyte recruitment to vascular walls and renal parenchyma [77,89,90,91]. TMAO activates the MAPK and NF-κB pathways in vascular cells, contributing to atherosclerotic inflammation and vascular calcification [92]. In transplant recipients, elevated circulating LPS correlates with increased pro-inflammatory CD14+CD16+ monocyte counts and reduced FoxP3+ Tregs—immunological correlates of allograft vulnerability and cardiovascular risk. Inflammaging—the chronic sterile low-grade inflammation that accelerates immunosenescence—is significantly promoted by dysbiosis-derived metabolites including uremic toxins and ammonia, compounding the immune dysfunction already induced by immunosuppressive therapy [12,77,92,93,94,95].

### 6.9. Hypertension

Post-transplant hypertension is nearly universal, affecting more than 80% of kidney transplant recipients, and is a major determinant of long-term cardiovascular mortality and graft loss. Beyond the well-established contributions of calcineurin inhibitors and corticosteroids to blood pressure elevation, gut dysbiosis provides a mechanistically distinct and clinically significant pathway to post-transplant hypertension. The depletion of SCFA-producing bacteria—particularly *Faecalibacterium*, *Roseburia*, and *Lachnospira*—reduces butyrate and propionate concentrations, impairing GPR41- and GPR43-mediated vascular tone regulation and diminishing eNOS-dependent NO bioavailability, resulting in impaired arterial [14,89,90,91].

TMAO, generated in excess by dysbiotic pathobionts from dietary choline and carnitine, suppresses endothelial NOS uncoupling through FoxO3a-mediated antioxidant pathway activation, reducing NO production and promoting oxidative stress in the vascular endothelium [77]. LPS-driven endotoxemia activates TLR4-NF-κB signaling to produce pro-inflammatory cytokines that increase endothelial VCAM-1 and ICAM-1 expression, promote arterial stiffness through leukocyte-mediated adventitial inflammation, and amplify angiotensin II-induced vasoconstriction. Dysbiosis in spontaneously hypertensive animal models is characterized by increased *Streptococcus*, *Ruminococcus gnavus*, and Proteobacteria alongside decreased Prevotella-9 and Bifidobacteriaceae—a pattern directly analogous to that observed in post-transplant dysbiosis [96,97]. The potential for microbiome-targeted interventions (dietary fiber supplementation, SCFA-producing probiotic strains) to complement antihypertensive pharmacotherapy in transplant recipients represents a clinically attractive but understudied avenue that warrants prospective investigation.

### 6.10. Quality of Life

Gut dysbiosis contributes to a broad spectrum of symptoms that collectively impair health-related quality of life (HRQoL) in renal transplant recipients. Gastrointestinal manifestations—including diarrhea, constipation, bloating, and cramping—arising from dysbiosis-induced barrier dysfunction and altered colonic motility constitute a major source of patient-reported disability [51]. These symptoms are further compounded by the adverse gastrointestinal profiles of immunosuppressive agents, creating a compound burden of GI morbidity that frequently leads to dose reductions with attendant risks of alloimmune events.

Systemic consequences of dysbiosis extend beyond the gut. Fatigue and weakness, attributable to the combined effects of uremic toxin accumulation, chronic inflammation, and anemia, are among the most prevalent complaints in KTRs and are closely linked to microbiome-driven IS and p-CS burden [77]. Dysbiosis-associated pruritus is mediated in part by IS- and p-CS-driven activation of PAR-2 receptors, while cognitive dysfunction—increasingly recognized in CKD and transplant populations—is promoted by dysbiosis-driven neuroinflammation secondary to LPS and TMAO crossing the blood–brain barrier and by depletion of neuroprotective SCFA metabolites [77,95]. The dysbiosis-driven cycles of urinary tract infection, C. difficile infection, and other infectious complications further disrupt daily functioning and mandate repeated hospitalizations. Taken together, these data demonstrate that restoration of a healthy gut microbiome, through dietary optimization, probiotic supplementation, or FMT, holds the potential not only to improve clinical endpoints but also to meaningfully enhance the HRQoL of kidney transplant recipients—a goal that should feature prominently in the design of future microbiome intervention trials [51,76].

## 7. Immunosuppressive Drugs and the Gut Microbiota: A Bidirectional Relationship

The relationship between immunosuppressive pharmacotherapy and the gut microbiota following renal transplantation is fundamentally bidirectional: each drug class shapes the intestinal microbial landscape in characteristic ways, while the resulting microbiota in turn modulates drug pharmacokinetics through enzymatic biotransformation, altered intestinal barrier permeability, and modulation of drug-metabolizing enzyme expression. Understanding these interactions is clinically important, as they may account for a substantial fraction of the inter-patient variability in immunosuppressive drug levels, efficacy, and toxicity observed in clinical practice [81,84].

### 7.1. Modification of Gut Microbiota by Immunosuppressive Drugs

Corticosteroids (prednisolone, methylprednisolone) are administered universally in the immediate post-transplant period and contribute to dysbiosis through multiple mechanisms. Glucocorticoid receptor activation in innate lymphoid cells and Th17 cells suppresses IL-22 production, impairing the maintenance of intestinal epithelial barrier integrity and reducing the synthesis of antimicrobial peptides (alpha- and beta-defensins, RegIII) that normally restrict pathobiont expansion (Sivaraj et al., 2020) [87]. Glucocorticoids additionally directly inhibit IgA secretion, further attenuating mucosal humoral defense. Studies in murine models demonstrate that prednisolone treatment enriches Proteobacteria and Enterobacteriaceae while depleting *Clostridium*, *Ruminococcus*, and members of the Lachnospiraceae family—taxa essential for SCFA production and colonization resistance [20,87].

Tacrolimus, the cornerstone calcineurin inhibitor in contemporary transplant immunosuppression, produces significant intestinal dysbiosis through both immunological and direct mucosal mechanisms. By blocking calcineurin-NFAT signaling, tacrolimus suppresses IL-2, IL-4, and IFN-γ production by T cells; crucially for intestinal homeostasis, it impairs IL-22 secretion from innate lymphoid cells and Th17 cells, reducing epithelial tight junction protein expression and antimicrobial peptide output [87]. Tacrolimus also increases intestinal epithelial cell apoptosis and reduces mucin production through goblet cell dysfunction, promoting barrier disruption. Clinically, tacrolimus-treated animals demonstrate increased Enterobacteriaceae and Streptococcaceae alongside decreased Lachnospiraceae and Ruminococcaceae; similar patterns have been identified in human KTR cohorts, with dysbiosis severity correlating with tacrolimus blood levels [20,51].

Cyclosporine, now used less frequently than tacrolimus, exerts broadly similar dysbiotic effects through calcineurin inhibition. However, its more pronounced direct epithelial cytotoxicity leads to higher intestinal epithelial apoptosis rates, compounding barrier disruption. Cyclosporine treatment enriches Proteobacteria, Enterobacteriaceae, and *Clostridium* while depleting beneficial commensals, and clinical studies confirm significant dysbiosis in cyclosporine-treated recipients [87]. The candidosis risk is heightened with cyclosporine compared to tacrolimus, reflecting distinct microbiome profiles; Candida spp. overgrowth is consistently reported in cyclosporine-treated cohorts [23].

Mycophenolate mofetil (MMF) induces the most severe post-transplant dysbiosis of all routinely used immunosuppressive agents. Beyond its immunosuppressive mechanism—inhibition of inosine monophosphate dehydrogenase (IMPDH) to block lymphocyte proliferation—MPA, the active form of MMF, exerts a direct antimicrobial effect against Gram-positive commensals, particularly members of the Firmicutes phylum including Lachnospiraceae and *Clostridium* clusters IV and XIVa. This selective bacteriostatic activity against protective commensals, while sparing Gram-negative organisms (which are intrinsically more resistant to IMPDH inhibition), creates a powerful selection pressure favoring expansion of *Escherichia*/*Shigella*, Proteobacteria, and other pathobionts [76,84,87]. Experimental data demonstrate that MMF exposure enriches lipopolysaccharide biosynthesis and beta-glucuronidase-producing pathways, further amplifying endotoxemia and drug-microbiota pharmacokinetic interactions [87].

The mTOR inhibitors sirolimus and everolimus produce a dysbiotic pattern broadly analogous to calcineurin inhibitors, characterized by enrichment of Enterobacteriaceae and Streptococcaceae with depletion of Lachnospiraceae. Mechanistically, mTORC1 inhibition impairs intestinal epithelial cell proliferation (for which mTOR signaling is required), reducing epithelial renewal and barrier integrity. Suppression of IL-22-driven barrier fortification and reduction in goblet cell mucin secretion further promote a leaky gut phenotype [20,87]. Notably, everolimus-treated recipients demonstrate distinct functional metagenomic signatures compared with tacrolimus-based regimens—including differences in carbohydrate metabolism gene representation and macrolide resistance markers—with clinical implications for infection susceptibility and drug-microbiota interactions [20]. Rapamycin has been shown to increase *Allobaculum*, *Bacteroides*, and *Lactobacillus* in some models, potentially reflecting its anti-inflammatory and autophagy-promoting properties, although depletion of *F. prausnitzii* remains a consistent finding across mTOR inhibitor-treated cohorts.

### 7.2. Influence of Gut Microbiota on Immunosuppressive Drug Metabolism

The gut microbiota influences the pharmacokinetics of immunosuppressive agents through multiple enzymatic and physiological mechanisms, with the net effect being substantial inter-patient variability in drug bioavailability and systemic exposure that cannot be fully explained by host genetic polymorphisms in drug-metabolizing enzymes alone [83,84].

For tacrolimus, gut microbial metabolism represents a clinically documented source of pharmacokinetic variability. Lee et al. (2015) made the landmark discovery that *Faecalibacterium prausnitzii* directly metabolizes tacrolimus to a less immunosuppressive metabolite M1, via a bacterial enzymatic activity that is heat-sensitive and therefore unambiguously of microbial origin [19]. Guo et al. subsequently demonstrated that M1 is approximately 15-fold less potent than tacrolimus at inhibiting peripheral blood mononuclear cell activation, and that this metabolic capacity is shared by many species within the Clostridiales order—a taxonomic group substantially depleted in post-transplant dysbiosis [98,99]. A pilot study confirmed that M1 is detectable in the blood of all 10 KTRs tested following oral tacrolimus administration, establishing that gut microbial metabolism of tacrolimus occurs in vivo and is clinically relevant [84]. Furthermore, the abundance of *F. prausnitzii* positively correlates with tacrolimus dosing requirements—patients with higher *F. prausnitzii* counts require higher oral doses to achieve target blood levels, consistent with greater first-pass microbial inactivation and intact P-glycoprotein function in a healthier gut [84].

Indirectly, dysbiosis suppresses intestinal CYP3A4 expression through LPS-TLR4-NF-κB signaling and through dysbiosis-driven pro-inflammatory cytokines (TNF-α, IL-1β, IL-6) that downregulate CYP3A4 transcription via JAK-STAT and NF-κB pathways—an effect that reduces the first-pass metabolism of tacrolimus, cyclosporine, and mTOR inhibitors, resulting in higher systemic drug exposure [76]. These dysbiosis-dependent elevations in drug bioavailability can manifest clinically as calcineurin inhibitor nephrotoxicity, neurotoxicity, or metabolic adverse effects, requiring dose adjustments that may be difficult to predict without microbiome information. By contrast, P-glycoprotein (P-gp) activity is impaired by dysbiosis-associated barrier dysfunction, further augmenting drug absorption. These combined effects on CYP3A4 and P-gp explain why dysbiotic transplant recipients frequently require lower tacrolimus and cyclosporine doses to maintain target blood levels.

For MMF, the impact of microbiota on drug metabolism is quantitatively the most significant of all immunosuppressants. Bacterial beta-glucuronidase (GUS) activity, expressed by commensal genera including *Bacteroides*, *Faecalibacterium*, and select Clostridiales, deconjugates MPAG to release active MPA in the colon, supporting enterohepatic recirculation and maintaining systemic MPA exposure. MMF-induced dysbiosis, by depleting GUS-expressing commensals while enriching pathobionts with low GUS activity, progressively impairs this enterohepatic reactivation, reducing total MPA bioavailability—potentially by 30–50% in states of severe dysbiosis—and creating a paradox in which the drug itself undermines its own pharmacokinetics [51,83,84]. This GUS-dependent mechanism has been validated in clinical practice: vancomycin (which reduces GUS-expressing bacteria) mitigates MMF-associated GI toxicity in animal models by reducing luminal MPA reactivation, while conversely, recipients with high GUS activity experience both greater MPA bioavailability and greater GI toxicity [83,84].

Corticosteroids undergo hepatic glucuronidation forming inactive metabolites, and bacterial GUS activity in the colon contributes to their enterohepatic recycling in a manner analogous to MMF, though quantitatively less prominent. Dysbiosis-driven reduction in commensal GUS activity may modestly reduce systemic steroid exposure through impaired enterohepatic recirculation, contributing to inter-patient variability in immunosuppressive [81]. For mTOR inhibitors, dysbiosis-induced CYP3A4 suppression is the dominant pharmacokinetic mechanism, producing increased sirolimus and everolimus bioavailability in the dysbiotic state—a pattern of parallel pathophysiology to the calcineurin inhibitors [76]. The broad clinical implication of these findings is that the intestinal microbiome should be considered as an important covariate when interpreting immunosuppressive drug level monitoring and designing dosing protocols, particularly in the early post-transplant period when dysbiosis is most dynamic and drug levels are most volatile.

## 8. Gut-Microbiota Therapies in Kidney Transplantation: The Gut–Liver–Kidney Axis Perspective

Throughout this review, findings are drawn from three tiers of evidence that differ in strength: (i) preclinical and mechanistic data from in vitro systems and animal models; (ii) observational human data from cross-sectional and cohort studies, which establish association but not causation; and (iii) interventional data from randomized controlled trials (RCTs). The majority of microbiome–kidney findings discussed here fall into the first two categories. Interventional evidence in chronic kidney disease is largely confined to small, short-duration trials using surrogate endpoints–for example, the randomized, placebo-controlled SYNERGY trial, in which synbiotic therapy lowered serum p-cresyl sulfate, with a less consistent effect on indoxyl sulfate and no improvement in measured kidney function [100]. In kidney transplant recipients specifically, microbiome-targeted interventions such as fecal microbiota transplantation have so far been evaluated only in case reports and small retrospective series rather than in controlled trials. Microbiome-targeted therapeutic strategies in chronic kidney disease and renal transplantation together with their proposed mechanisms of action and the current level of supporting evidence are briefly summarized in Table 1. Statements regarding clinical benefit should therefore be regarded as provisional pending adequate evaluation.

### 8.1. Dietary Interventions

Dietary modification represents the most accessible and physiologically rational approach to modulating the gut microbiome in kidney transplant recipients. The composition and metabolic output of the intestinal microbiota are profoundly responsive to dietary substrate availability, and nutritional strategies that increase the delivery of fermentable fiber, polyphenols, and other prebiotic compounds to the colon can selectively promote the growth of SCFA-producing commensals while suppressing proteolytic pathobionts. The following subsections review the current evidence for specific dietary interventions [101].

#### 8.1.1. Fiber, Phytochemicals, and Fermented Foods

Dietary fiber is the foremost substrate for SCFA-producing commensal bacteria and a cornerstone of microbiome-targeted nutritional therapy. Epidemiological data consistently demonstrate an inverse association between dietary fiber intake and CKD progression, cardiovascular events, and mortality [70]. Clinical interventions utilizing resistant starch, inulin, and other prebiotic fibers have shown reductions in circulating uremic toxins (indoxyl sulfate and p-cresyl sulfate), improvements in inflammatory markers, and favorable shifts in microbiome composition in CKD and dialysis patients [70,102,103].

Phytochemicals—biologically active compounds found in fruits, vegetables, legumes, and whole grains—exert prebiotic-like effects by selectively promoting the growth of beneficial taxa while inhibiting pathogens. Polyphenols, abundant in berries, tea, and dark chocolate, undergo extensive microbial biotransformation in the colon and have demonstrated anti-inflammatory, antioxidant, and renoprotective properties in preclinical models. Fermented foods (yogurt, kefir, sauerkraut, kimchi, kombucha) provide live microorganisms that may transiently colonize the gut, enhance microbial diversity, and modulate immune responses. However, caution is warranted in CKD patients, as some fermented foods are high in sodium, potassium, and phosphorus [101,104].

Plant-based dietary patterns, incorporating at least 30 diverse plant foods per week, have been associated with enhanced gut microbiome diversity, increased butyrate production, reduced dietary acid load, and improved constipation in CKD cohorts. Concerns regarding hyperkalemia with plant-based diets in CKD appear to be less clinically significant than previously assumed, as the lower bioavailability of potassium from plant sources and the alkalinizing effect of these diets may offset the risk [105].

#### 8.1.2. Traditional Chinese Medicine (TCM)

Traditional Chinese Medicine has a long history of use in kidney disease, and recent research has provided mechanistic insights into the microbiome-mediated effects of several TCM formulations. TCM preparations, including Qing-Re-Xiao-Zheng formula, Shenyan Kangfu tablets, Huangkui capsules, and Jian-Pi-Yi-Shen Formula, have been shown to modulate the gut microbiota by restoring the balance of beneficial bacteria (e.g., *Lactobacillus*, *Bifidobacterium*) while reducing pathogenic taxa (e.g., Escherichia, Enterococcus). These interventions have demonstrated reductions in circulating uremic toxins, attenuation of intestinal barrier dysfunction, and amelioration of renal inflammatory and fibrotic pathways in preclinical models of diabetic kidney disease and CKD. While the evidence base for TCM in this context is growing, most studies remain preclinical or of limited sample size, and rigorous randomized controlled trials are needed to establish efficacy and safety in the clinical setting [106,107,108].

### 8.2. Bile Acid–Based Therapeutic Strategies

Given the central role of bile acid signaling in the gut–liver–kidney axis, pharmacological modulation of bile acid receptors has emerged as a therapeutic strategy. FXR agonists, such as obeticholic acid, restore intestinal barrier function, reduce bacterial translocation, suppress hepatic inflammation, and have shown renoprotective effects in preclinical CKD models. TGR5 agonists promote GLP-1 secretion, enhance energy expenditure, and exert anti-inflammatory effects in the kidney. Bile acid sequestrants (e.g., sevelamer, used clinically as a phosphate binder) may additionally modulate bile acid metabolism with potential downstream benefits for the gut microbiota and renal function [109,110,111].

### 8.3. Physical Adsorbents, Probiotics, Prebiotics, and Synbiotics

#### 8.3.1. Physical Adsorbents

Oral adsorbents such as AST-120 (spherical carbon adsorbent) are designed to bind uremic toxin precursors (e.g., indole) in the gastrointestinal tract, preventing their absorption and subsequent conversion to indoxyl sulfate. Clinical trials in Japan and other Asian countries have demonstrated reductions in serum indoxyl sulfate and modest attenuation of CKD progression with AST-120 use, although results from the large multinational EPPIC trials were inconclusive, highlighting challenges in patient selection and study design [112].

#### 8.3.2. Probiotics

Probiotics—defined as live microorganisms that, when administered in adequate amounts, confer a health benefit on the host—have been extensively investigated in CKD and dialysis populations. Specific strains of *Lactobacillus* and *Bifidobacterium* have demonstrated the ability to reduce circulating levels of uremic toxins (indoxyl sulfate, p-cresyl sulfate), decrease inflammatory biomarkers (TNF-α, IL-6, CRP), and alleviate gastrointestinal symptoms in hemodialysis and peritoneal dialysis patients. A systematic review published in 2024 confirmed significant positive effects of probiotics on gut dysbiosis, uremic toxin levels, inflammation, and quality of life in dialysis patients [113].

#### 8.3.3. Prebiotics and Synbiotics

Prebiotics—non-digestible substrates (e.g., inulin, fructo-oligosaccharides, galacto-oligosaccharides) that selectively stimulate the growth of beneficial bacteria—offer a complementary approach to probiotics. Network meta-analyses have demonstrated that prebiotics are superior for reducing specific inflammatory markers (IL-6, TNF-α) and uremic toxins (indoxyl sulfate), while synbiotics (combined pre- and probiotics) are more effective for reducing CRP and endotoxin levels. A Cochrane Review (2023) cautioned that current evidence remains of low to moderate certainty, and large-scale, well-designed trials are needed to provide definitive clinical recommendations [114].

### 8.4. Fecal Microbiota Transplantation (FMT)

Fecal microbiota transplantation—the transfer of processed stool from a healthy donor to a recipient’s gastrointestinal tract—represents the most direct approach to restoring microbial diversity and function. Originally established for the treatment of recurrent Clostridioides difficile infection, FMT is now being explored in a range of conditions characterized by gut dysbiosis, including CKD [115].

A 2024 clinical trial demonstrated that significantly fewer patients in the FMT group (13.3%) progressed to advanced CKD compared with the placebo group (53.8%), with stable renal function parameters including serum creatinine and urea nitrogen. Additional prospective studies have reported a reduction in proteinuria, improvement in gastrointestinal symptoms, and a response rate of approximately 41% in CKD patients treated with FMT. Mechanistically, FMT is hypothesized to restore intestinal barrier integrity, attenuate systemic inflammation, inhibit renin-angiotensin system activation, and remodel the gut microbiota toward a healthier composition. Preclinical and preliminary clinical data also support the application of FMT in CKD subtypes including diabetic nephropathy, IgA nephropathy, membranous nephropathy, and focal segmental glomerulosclerosis [116].

Despite these encouraging results, significant challenges remain. Most FMT studies in CKD are small and uncontrolled, donor selection and stool preparation protocols are not standardized, and long-term safety data are limited. Concerns regarding the transmission of multidrug-resistant organisms, opportunistic infections in immunosuppressed transplant recipients, and regulatory barriers must be addressed before FMT can be recommended as a routine clinical intervention. 

### 8.5. The Diet–Microbiota–Drug Triad

The concept of the diet–microbiota–drug triad recognizes the interplay among dietary habits, microbial metabolism, and pharmacological interventions as a key determinant of treatment outcomes. In CKD and transplant recipients, immunosuppressive agents (e.g., tacrolimus, mycophenolate mofetil) are themselves metabolized by or interact with the gut microbiota, and microbiome composition may influence drug bioavailability, efficacy, and toxicity. Conversely, these drugs alter microbial community structure, potentially exacerbating dysbiosis. An integrated approach that considers dietary patterns and microbiome status alongside pharmacotherapy may therefore optimize therapeutic outcomes and reduce adverse effects [117,118,119].

### 8.6. Novel Drug Approaches

Beyond established microbiome-targeted interventions, several novel pharmacological strategies are under development. Targeted inhibitors of microbial enzymes—such as TMA lyase inhibitors (e.g., 3,3-dimethyl-1-butanol) that block TMAO production, and tryptophanase inhibitors that reduce indole and indoxyl sulfate generation—offer the prospect of selectively modulating the metabolic output of the gut microbiota without disrupting its overall composition. Engineered probiotics, designed using synthetic biology approaches including CRISPR-based systems, represent an emerging frontier; these “smart bacteria” can be programmed to sense disease-related signals, produce therapeutic molecules, or degrade pathogenic metabolites within the gut. Postbiotics—bioactive compounds derived from probiotic organisms through fermentation processes—and microbiome-derived small molecules are also being explored as safer alternatives to live microbial interventions [118,119].

## 9. Clinical Significance and Therapeutic Approaches

### 9.1. Effects of Probiotics, Prebiotics, and Synbiotics on Patients with CKD

The clinical application of probiotics, prebiotics, and synbiotics in CKD encompasses a range of endpoints including reduction in uremic toxins, attenuation of systemic inflammation, improvement of gastrointestinal symptoms, and potentially slowing of disease progression. Meta-analyses of randomized controlled trials have demonstrated modest but significant reductions in serum p-cresyl sulfate, indoxyl sulfate, blood urea nitrogen, and inflammatory biomarkers with these interventions. Specific probiotic formulations containing *Lactobacillus acidophilus*, *Bifidobacterium longum*, and *Streptococcus thermophilus* have shown particular promise in reducing uremic toxin burden and improving quality of life [25,120,121,122].

However, clinical evidence remains heterogeneous, with variability in probiotic strains, dosing regimens, duration of treatment, and patient populations limiting the generalizability of results. A Cochrane Review concluded that there is currently insufficient high-certainty evidence to definitively support or refute the routine use of these interventions in CKD, underscoring the need for large-scale, methodologically rigorous trials [27,109,123].

### 9.2. Application in ESRD Patients

In patients with ESRD receiving hemodialysis or peritoneal dialysis, the gut microbiota is subject to particularly severe disruption due to the confluence of uremia, restricted dietary intake, frequent antibiotic use, and altered gastrointestinal transit. Probiotics and synbiotics have been shown to reduce protein-bound uremic toxins not efficiently cleared by dialysis, decrease inflammatory markers, and improve gastrointestinal complaints in this population. The reduction in endotoxemia through restoration of intestinal barrier function may additionally mitigate the chronic inflammatory state that drives cardiovascular morbidity and mortality in the dialysis population [121,123].

### 9.3. Translational Potential of Plant-Based Diets in Peritoneal Dialysis and Hemodialysis Patients

Plant-based diets are increasingly advocated for CKD and dialysis patients based on their capacity to deliver high-fiber, low-acid-load nutritional profiles that support a diverse, SCFA-producing gut microbiota. Clinical studies in peritoneal dialysis and hemodialysis cohorts have demonstrated associations between higher plant food consumption and improved inflammatory profiles, lower uremic toxin levels, and better patient-reported outcomes. The integration of plant-based dietary counseling into the routine care of dialysis patients, with appropriate monitoring for hyperkalemia and nutritional adequacy, represents a pragmatic and low-risk therapeutic strategy [27,104,124,125].

### 9.4. Mechanisms and Clinical Translation of FMT in Dialysis Patients

The application of FMT in the dialysis population remains largely investigational but offers the theoretical advantage of comprehensive microbiome reconstitution. Preclinical data support the ability of FMT to restore microbial diversity, reduce uremic toxin production, and attenuate systemic inflammation in uremic animal models. Clinical translation, however, requires resolution of substantial practical and safety concerns, including the risk of infection in immunocompromised patients, the need for standardized donor screening and stool processing protocols, and the absence of regulatory frameworks governing FMT use in CKD and transplantation [116,119,126,127].

## 10. Prospects and Summary

### 10.1. Challenges in Clinical Application and Personalized Medicine

Despite the compelling mechanistic rationale for microbiome-targeted therapies in CKD and renal transplantation, several challenges impede their clinical translation. The high inter-individual variability in gut microbiome composition, dietary habits, genetic background, and medication use complicates the identification of universally effective interventions. The absence of standardized analytical methods for microbiome profiling—including sample collection, DNA extraction, sequencing platforms, and bioinformatic pipelines—limits comparability across studies and reproducibility of findings. Furthermore, the interplay between the microbiome and immunosuppressive agents in transplant recipients introduces an additional layer of complexity that is only beginning to be explored.

Personalized medicine approaches, leveraging multi-omics profiling (metagenomics, metabolomics, proteomics, immunophenotyping) and machine learning algorithms, hold the promise of identifying patient-specific microbiome signatures that predict treatment response and enable tailored therapeutic strategies. The integration of artificial intelligence (AI) into microbiome research may facilitate the design of personalized probiotic cocktails, dietary prescriptions, and FMT protocols optimized for individual patients.

### 10.2. From Mechanism to Practice: Clinical Evidence

The transition from mechanistic insight to clinical implementation requires robust evidence from large-scale, multicenter, randomized controlled trials with well-defined clinical endpoints. While a substantial body of preclinical and pilot clinical data supports the therapeutic potential of microbiome modulation in CKD and transplantation, many studies are limited by small sample sizes, heterogeneous designs, and short follow-up periods. Registries, biobanks, and prospective cohort studies that integrate longitudinal microbiome profiling with clinical outcomes data will be essential for advancing the evidence base.

### 10.3. Future Directions and Research Priorities

Several high-priority areas for future investigation can be identified. First, clinical trials evaluating FMT in renal transplant recipients, with attention to safety in the context of immunosuppression, are urgently needed. Second, the development and validation of microbiome-based biomarkers for early detection of graft rejection and CKD progression should be accelerated. Third, studies elucidating the interactions between immunosuppressive drugs and the gut microbiota—including the effects of tacrolimus, mycophenolate, and corticosteroids on microbial composition and metabolic function—may inform the design of microbiome-sparing immunosuppressive protocols. Fourth, the potential of CRISPR-engineered probiotics and targeted enzyme inhibitors to selectively modulate harmful metabolic pathways without disrupting overall microbial ecology warrants further preclinical and clinical evaluation. Finally, the integration of patient-reported outcomes—including gastrointestinal symptoms, dietary satisfaction, and quality of life—into the assessment of microbiome-targeted therapies will ensure that these interventions meet the needs and preferences of patients.

### 10.4. Limitations of the Current Evidence Base

A number of limitations temper the conclusions that can currently be drawn from microbiome research in chronic kidney disease and renal transplantation. Foremost is the pronounced inter-individual variability of the gut microbiome. Its composition is shaped by host genetics, age, sex, diet, geography, physical activity, comorbidities, and concomitant medications—including antibiotics, proton-pump inhibitors, and, in transplant recipients, immunosuppressive agents—such that no single “healthy” or “normal” reference community exists. This variability, compounded by the modest sample sizes and predominantly cross-sectional design of many studies, limits statistical power, hampers the identification of reproducible disease-associated signatures, and constrains the generalizability of findings across populations.

Second, methodological heterogeneity impedes comparison across studies. Results depend heavily on pre-analytical and analytical choices, including stool collection and storage, DNA extraction protocol, the sequencing strategy (16S rRNA amplicon sequencing versus shotgun metagenomics), the hypervariable region targeted, sequencing depth, and the bioinformatic pipeline, reference database, and taxonomic classifier applied. Most studies report relative rather than absolute abundances, which can distort inferred changes, and batch effects together with inconsistent reporting standards further reduce reproducibility. Apparently discordant findings between cohorts may therefore reflect technical rather than biological differences.

Third, establishing causality remains a central challenge. The majority of human data are observational and associative, and the direction of the relationship is frequently ambiguous: dysbiosis may drive kidney injury, but the uremic milieu, dietary restriction, and polypharmacy that characterize advanced chronic kidney disease may themselves reshape the microbiome, raising the possibility of reverse causation and residual confounding. Much mechanistic insight derives from gnotobiotic and antibiotic-treated rodent models whose translation to humans is uncertain. Stronger causal inference will require adequately powered interventional trials, longitudinal sampling, gnotobiotic and fecal-transfer experiments, and complementary approaches such as Mendelian randomization.

Finally, there are as yet no standardized microbiome-based therapeutic protocols. Probiotic, prebiotic, and synbiotic studies employ heterogeneous strains, formulations, doses, and durations, while FMT studies differ in donor selection and screening, material preparation, and the route and frequency of administration. Endpoints are similarly inconsistent, and much of the clinical literature relies on surrogate markers—such as circulating uremic-toxin concentrations—rather than hard clinical outcomes. The durability of microbiome modification, its long-term safety (particularly the risk of transmissible infection in immunosuppressed transplant recipients), and the regulatory pathway for these interventions all remain incompletely defined. Together, these gaps explain why mechanistically promising strategies have not yet translated into routine clinical practice, and they frame the research priorities outlined in Section 10.3.

## 11. Conclusions

The gut microbiome occupies a central position in the pathogenesis of chronic kidney disease and profoundly influences outcomes following renal transplantation. Through the interconnected gut–kidney, gut–liver, and liver–kidney axes, microbially derived metabolites modulate inflammation, fibrosis, immune regulation, and vascular integrity in ways that are both mechanistically compelling and clinically consequential. The identification of SCFAs, bile acids, TMAO, and tryptophan-derived uremic toxins as key mediators of inter-organ crosstalk has illuminated novel diagnostic and therapeutic opportunities. As detailed in this review, the impact of dysbiosis on kidney transplant outcomes is remarkably broad—encompassing allograft function and rejection risk, infection susceptibility, post-transplant diarrhea and diabetes, chronic inflammation, hypertension, and health-related quality of life—while the bidirectional pharmacokinetic interactions between immunosuppressive drugs and the gut microbiota add an additional layer of complexity that is only now beginning to be appreciated clinically.

A diverse arsenal of microbiome-targeted interventions—ranging from dietary modification and prebiotics to FMT and engineered probiotics—is now available or under active development, offering realistic prospects for improving the care of CKD and transplant patients. However, the field faces significant challenges in standardization, personalization, and the generation of high-quality clinical evidence. As the understanding of microbiome–host interactions deepens and technological platforms mature, we anticipate a paradigm shift toward personalized, microbiome-informed management strategies that will ultimately improve graft survival, reduce complications, and enhance the quality of life of patients with renal disease.

## Figures and Tables

**Table 1:** Microbiome-targeted therapeutic strategies in chronic kidney disease and renal transplantation: proposed mechanisms and current level of supporting evidence.

Therapeutic Strategy	Proposed Mechanism	Highest Current Level of Evidence	Key Considerations/Caveats
Dietary fiber and phytochemicals	Fermentable fiber ↑ SCFA-producing taxa; ↑ SCFAs promote Treg induction, epithelial barrier integrity and anti-inflammatory signaling; ↓ generation of protein-bound uremic toxins	Observational cohorts and small RCTs in CKD/dialysis (surrogate endpoints)	Low-risk, pragmatic; monitor for hyperkalaemia and nutritional adequacy
Plant-based diet	High-fiber, low-acid-load profile supporting a diverse, SCFA-producing microbiota; ↓ uremic toxin generation, improved inflammatory profile	Observational studies + small interventional trials in PD/HD cohorts	Requires potassium and protein-adequacy monitoring in advanced CKD
Fermented foods	Modulation of microbial composition; delivery of anti-inflammatory metabolites and live cultures	Preclinical + limited clinical data	Heterogeneous products; effects not standardized
Traditional Chinese Medicine (TCM)	Prebiotic-like modulation of microbial composition and metabolite output	Preclinical, with limited clinical data	Variable formulations; mechanisms incompletely defined
Bile-acid–based strategies (FXR/TGR5 modulation)	Regulation of renal lipid metabolism, fibrosis and inflammation via bile-acid receptor signaling	Preclinical (largely rodent models)	No dedicated transplant-population trials
Physical adsorbents (e.g., AST-120)	Luminal sequestration of indole and p-cresol precursors, lowering serum indoxyl sulfate and p-cresyl sulfate	RCTs in CKD (surrogate endpoints; neutral on hard renal endpoints in EPPIC trials)	Effect on clinical progression not demonstrated
Probiotics	Restoration of commensal balance; ↓ uremic toxin production and systemic inflammation (e.g., *L. acidophilus*, *B. longum*, *S. thermophilus*)	RCTs and meta-analyses in CKD/ESRD (surrogate endpoints); low-certainty per Cochrane review	Strain, dose and duration heterogeneity; no transplant-endpoint data
Prebiotics and synbiotics	Substrate for beneficial taxa; ↑ SCFA production; ↓ protein-bound uremic toxins	Small RCTs in CKD/dialysis (e.g., SYNERGY: ↓ p-cresyl sulfate; surrogate endpoints)	Inconsistent effect on indoxyl sulfate and measured kidney function
Fecal microbiota transplantation (FMT)	Wholesale reconstitution of microbial ecology; restored diversity, ↓ uremic toxin production, ↓ inflammation	Case reports/small retrospective series in transplantation; preclinical in uremic models	Infection risk under immunosuppression; donor-screening, processing and regulatory frameworks unstandardized
Engineered probiotics and enzyme inhibitors	Targeted suppression of harmful microbial pathways (e.g., TMA-lyase inhibition) without disrupting overall ecology	Preclinical only	Early stage; clinical safety and efficacy unestablished

## Data Availability

The original contributions presented in this study are included in the article. Further inquiries can be directed to the corresponding author.

## Abbreviations

The following abbreviations are used in this manuscript:

AhR	aryl hydrocarbon receptor
BCAAs	branched-chain amino acids
CKD	chronic kidney disease
DAMPs	damage-associated molecular patterns
DKD	diabetic kidney disease
ESRD	end-stage renal disease
FMT	fecal microbiota transplantation
FMO3	flavin-containing monooxygenases
FXR	farnesoid X receptor
GUS	beta-glucuronidase
HDACs	histone deacetylases
HRQoL	health-related quality of life
IDO	indoleamine 2,3-dioxygenase
IMPDH	inosine monophosphate dehydrogenase
IS	indoxyl sulfate
KTRs	kidney transplant recipients
KT	kidney transplantation
LPS	lipopolysaccharide
MMF	mycophenolate mofetil
MPA	Mycophenolic acid
MPAG	mycophenolic acid glucuronide
NF-κB	nuclear factor-kappa B
NODA	New-onset diabetes after transplantation
PAGln	phenylacetylglutamine
PAMPs	pathogen-associated molecular patterns
PTD	Post-transplant diarrhea
ROS	reactive oxygen species
SCFAs	short-chain fatty acids
TCM	Traditional Chinese Medicine
TGR5	G-protein-coupled receptors
TMAO	trimethylamine N-oxide
TMA	Trimethylamine
TLR	Toll-like receptor
UTIs	urinary tract infections
